# Investigating Menstrual Hygiene Behaviors: Analysis of Demographic and Social Factors Among Adolescent School Girls

**DOI:** 10.7759/cureus.70799

**Published:** 2024-10-03

**Authors:** Saraladevi Manimaran, Ramya Ramanathan, Sundari Subramanian

**Affiliations:** 1 Department of Pediatrics, Sree Balaji Medical College and Hospital, Chennai, IND

**Keywords:** adolescent girls, behavior analysis, demographic factors, menstrual hygiene, social factors

## Abstract

Introduction: This study examines the intricate relationship between demographic, social, and contextual factors that impact the behaviors related to menstruation hygiene among adolescent females, namely those between the ages of 13 and 15. Significant relationships are discovered between age groups, religious beliefs, family structures, maternal education levels, and sources of menstrual health information. These findings shed light on the many elements that influence menstrual health, with Hinduism being the most prevalent religion, followed by Christianity and Islam. Maternal education has a significant impact on nuclear families, highlighting the importance of mothers in menstrual health practices. Peer influence, teacher supervision, and social media play a significant role in shaping attitudes toward menstrual health, highlighting the need for customized educational interventions. The importance of infrastructure is underscored by the presence of easily accessible sanitation facilities, especially private bathrooms.

Methods: We conducted a thorough survey among adolescent females and performed statistical analysis, including correlation and regression. The focus groups yielded qualitative insights that offered further contextual information.

Results: There is a strong influence of demographic, social, and contextual factors on menstrual hygiene behaviors. Religious connections, maternal education, and access to sanitation influence menstrual health behaviors. The impact of peer influence and social media necessitates the implementation of customized instructional strategies.

Conclusion: Customized interventions and educational programs are crucial for ensuring positive menstruation experiences among adolescent girls. Longitudinal studies are essential for evaluating the effects of interventions. Utilizing community-based and digital health platforms can strengthen menstrual health, emphasizing the public health importance of menstruation health.

## Introduction

Adolescence is a significant phase recognized by the World Health Organization (WHO) that marks the transition from childhood to adulthood, between 10 and 19 years of age. Adolescence is a stage characterized by significant changes in the body, mind, and emotions, usually initiated by the onset of puberty and the start of menstruation in females. Menstruation is a crucial aspect of a woman's reproductive cycle. The significance of menstrual hygiene (MH) is emphasized, as defined by the United Nations International Children's Emergency Fund (UNICEF) as the appropriate handling of menstrual blood using appropriate sanitary products, in addition to practicing personal hygiene [1].

Failure to follow proper practices can lead to significant health issues, including reproductive tract infections and gynecological problems, underscoring the importance of menstrual hygiene. Adolescents, particularly in regions like India, face unique challenges in maintaining their mental well-being due to cultural beliefs and societal norms. Limited understanding of menstruation, along with societal limitations, often hinders open discussions and perpetuates the dissemination of inaccurate information [2]. Despite being a natural occurrence, menstruation still faces social disapproval and misinformation, especially among teenage girls. The limited understanding of this subject not only affects one's physical health but also plays a role in psychological and social challenges. Disparities in education and resources exacerbate the problem, especially in rural areas.

Government initiatives and health education interventions have made significant efforts to address mental health issues, given their substantial impact on an individual's overall well-being and development. However, there are still variations that persist, particularly between rural and urban areas, necessitating further examination and targeted interventions. Understanding the intricate and varied aspects of mental health is crucial. The impact of poor mental health practices extends beyond physical health risks like respiratory tract infections (RTIs) and gynecological complications. It also affects education and economic opportunities, particularly in developing countries such as India [3]. The lack of open discussion surrounding menstruation due to cultural and societal norms hinders the exchange of accurate information on the subject.

Government initiatives and health education programs have made efforts to address these challenges. However, significant disparities persist, particularly in remote regions with limited access to resources and educational opportunities. To tackle these disparities, it is essential to implement targeted measures that are tailored to meet the unique needs and circumstances of teenage girls, especially those residing in marginalized areas [2,3]. This study seeks to evaluate the effectiveness of health education interventions in enhancing menstrual hygiene (MH) among adolescent girls in urban schools in Chennai. Through a thorough evaluation, the goal is to enhance understanding and promote sustainable behaviors that support menstrual health and well-being. This research seeks to address the existing gap in the literature and offer valuable insights into the efficacy of interventions that empower adolescent girls to effectively manage their menstrual health [3].

## Materials and methods

Study design

The study employs a semi-structured questionnaire to gather data on demographic information, parental education, age at menarche, and knowledge, attitudes, and practices associated with menstruation. We will obtain written consent before administering the questionnaire. The analysis will focus on understanding knowledge, attitudes, and behaviors related to mental health, as well as the different factors associated with it. Methods to evaluate the face validity and reliability of the data will be utilized (Table 1).

**Table 1:** Parameters for sample collection.

Study design	Cross-sectional study
Study place	Government and private schools in Pallavaram taluk
Study population	Adolescent girls of 10–19 years in the government and private schools in Pallavaram taluk
Sampling method	Simple Random sampling
Study period	Six months

Study setting

The study will be conducted in Pallavaram taluk, encompassing both government and private schools.

Study population

The target population includes adolescent girls aged 10 to 19 years who are enrolled in schools within Pallavaram taluk.

Sample size calculation

Determining the sample size for studying school-aged girls' MH practices in Pallavaram involves Dobson's formula. With a population size of 10,000, the aim is to estimate pad usage proportion with a 95% confidence level and a margin of error of 0.05. Utilizing Dobson's formula ensures statistical precision in estimating population proportions [4,5]. This approach enables researchers to design studies that yield reliable and representative findings regarding MH practices among school-aged girls, contributing to evidence-based interventions and policies.

Sampling framework

A comprehensive list of schools within Pallavaram Taluk will be compiled with permission obtained from the Department of Public Instruction (DPI). Excluded from this list will be nursery schools and those catering exclusively to boys. Random numbers will then be assigned to the shortlisted schools using a random number generator or Microsoft Excel's (Microsoft Corporation, Redmond, Washington, USA) random number function. Adolescent girls aged 10 to 19 from selected schools will be informed about the study, and written consent will be obtained prior to data collection. Information will be collected through a semi-structured questionnaire, and subsequent analysis will focus on understanding knowledge, attitudes, and behaviors related to MH, along with associated factors.

Tool development and description

We rigorously developed the questionnaire for this study, incorporating insights from literature reviews and expert consultations. We designed the questionnaire in Tamil to align with the linguistic preferences of the target population, aiming to gather demographic data and evaluate the knowledge, attitudes, and practices (KAP) associated with MH among adolescent girls.

Data collection and analysis

The data collection process involved distributing the questionnaire to adolescent girls, followed by a comprehensive educational program on MH. After a six-month interval, we conducted a follow-up survey to assess changes in KAP regarding MH. Data analysis was conducted using IBM SPSS Statistics for Windows, Version 20 (Released 2011; IBM Corp., Armonk, New York, USA), with demographic data presented using frequencies and percentages. We assessed associations between variables using the Chi-square test and provided visual representations using bar charts and pie charts. We set the significance level at 0.05 [6].

## Results

Sample size calculation

The individual participants were selected according to the predetermined sample size calculated using Dobson's formula. The estimation of the proportion (P) of girls using disposable pads, based on existing data or preliminary investigations, led to P = 0.37 and Q = 0.63. Employing a margin of error (d) of 0.05 (5%) for a desired precision level of 5%, Dobson's formula computed the required sample size (n) of approximately 359 adolescent girls. Utilizing this formula, the calculation yielded n ≈ 359, ensuring a 95% confidence level in estimating the proportion of girls using disposable pads with a margin of error of 5%. Therefore, individual participants were selected from the enrolled girls within the selected schools to meet the required sample size, ensuring adequate statistical power for the study.

Age composition of the participants

The majority of participants fall within the age range of 13-15 years, constituting 74.93% of the total sample size. Adolescents aged 10-12 years represent a smaller proportion, accounting for 20.61% of the participants. A minority of participants, comprising 4.46% of the total sample, fall within the age group of 16-18 years. Overall, the sample size of 359 participants captures a diverse representation of adolescents across different age brackets (Table 2).

**Table 2:** Distribution of participants by age group. N: number, %: percentage; Chi-square test result: χ² = 186.4, df = 4, p < 0.001 (considered significant, if p < 0.05 to p < 0.001).

Age group	Frequency (N)	Percentage (%)
10-12	74	20.61
13-15	269	74.93
16-18	16	4.46
Total	359	100

Religious composition of the participants

The Hindu community constitutes the largest religious group among the participants, comprising 74.37% (267/359) of the total sample size. Christians represent the second most prevalent religious affiliation, with a proportion of 17.83% (64/359). Muslims constitute a smaller yet significant portion of the sample, comprising 7.80% (28/359) of the total participants. Collectively, these findings reflect the religious diversity present within the study population, providing valuable insights into the cultural and demographic landscape of the community under investigation (Table 3).

**Table 3:** Distribution of participants by religion. N: number; %: percentage; Chi-square test result: χ² = 24.3, df = 2, p < 0.001 (considered significant, if p < 0.05 to p < 0.001).

Religion	Frequency (N)	Percentage (%)
Hindu	267	74.37
Christian	64	17.83
Muslim	28	7.80
Total	359	

Distribution of participants by family type

The majority of participants belong to nuclear families, constituting 85.24% (306/359) of the total sample size. Conversely, a smaller proportion of participants are from joint families, comprising 14.76% (57/359) of the total sample. This distribution provides insights into the prevailing family structures within the study population, shedding light on the social dynamics and familial contexts that may influence adolescent behaviors and perceptions (Table 4).

**Table 4:** Distribution of participants by family type. Analysis: The data has been represented as N, %; Chi-square test result: χ² = 109.2, df = 8, p < 0.001 (p-value is considered significant if p < 0.05, p < 0.001). There is a significant association between sources of knowledge on MH among participants (χ² = 109.2, p < 0.001). The distribution of knowledge sources varies significantly among the participants. MH: menstrual hygiene.

Type of family	Frequency (N)	Percentage (%)
Nuclear	306	85.24
Joint	53	14.76
Total	359	

Educational status of mother

The majority of mothers have attained education up to the higher secondary level, constituting 46.52% (167/359) of the total sample size. Following this, a significant proportion of mothers have completed education up to the high school level, comprising 31.48% (113/359) of the sample (Table 5). A smaller percentage of mothers have pursued further education up to the degree level, representing 17.83% (64/359) of the total participants. Additionally, a very small proportion of mothers are either illiterate or have received education up to primary and middle school levels, accounting for 0.28% (1/359) and 3.90% (14/359) of the sample, respectively. 

**Table 5:** Educational status of mothers. Analysis: The data has been represented as N, %; Chi-square test result: χ² = 75.6, df = 8, p < 0.001 (considered significant if p < 0.05 to p < 0.001). There is a significant association between the educational status of mothers and other variables (χ² = 75.6, p < 0.001). The distribution of educational status among mothers differs significantly among the categories.

Educational status of mother	Frequency (N)	Percentage (%)
Illiterate	1	0.28
Primary and middle school	14	3.90
High school	113	31.48
Higher secondary	167	46.52
Degree	64	17.83

Sources of knowledge on MH among participants

The primary source of knowledge reported by participants was maternal guidance, with 162 respondents accounting for 45.13% of the total sample. This underscores the significant role mothers play in imparting information and practices related to MH to their daughters. Friends were identified as another notable source of knowledge, with 84 respondents indicating reliance on peers for MH information, constituting 23.4% of the sample. The influence of friends highlights the importance of peer networks in disseminating relevant information and fostering supportive environments. Teachers were cited as a source of knowledge by 54 participants, representing 15.04% of the sample. This emphasizes the role of educational institutions in providing structured education on MH and related health topics. Furthermore, an equal number of participants (54) reported acquiring knowledge from social media platforms. This finding underscores the growing influence of digital media in disseminating health-related information, including MH practices (Table 6).

Interestingly, a relatively small proportion of respondents (five participants, 1.39%) cited books as their source of knowledge on MH. While books remain a traditional source of information, their limited prevalence in this study suggests a shift towards more interactive and accessible sources. These findings underscore the importance of employing diverse educational strategies and platforms to disseminate accurate and comprehensive information on MH.

**Table 6:** Sources of knowledge on MH among participants. Analysis: The data has been represented as N, %; Chi-square test result χ² = 7.9, df = 8, p < 0.001 (considered significant if p < 0.05 to p < 0.001). There is a significant association between the knowledge acquired from variables (χ² = 7.9, p < 0.001). MH: menstrual hygiene.

Knowledge	Frequency (N)	Percentage (%)
Mother	162	45.13
Friends	84	23.4
Teacher	54	15.04
Social media	54	15.04
Books	5	1.39

Sanitization facility distribution among study participants

Types of facilities along with their corresponding frequencies and percentages. The majority of participants reported having access to individual residence restrooms, constituting 266 out of 359 participants and representing 74.09% of the sample. Multi-family restrooms were also commonly reported, with 73 participants indicating their usage, accounting for 20.33% of the sample. A small proportion of participants reported utilizing public toilets, comprising only one respondent, which translates to 0.28% of the sample. Additionally, 19 participants reported having more than one restroom in their homes, representing 5.29% of the total sample population (Table 7).

**Table 7:** Sanitization facility distribution in the population. Analysis: The data has been represented as N, and percentage (%).

Types of sanitization facility	Frequency (N)	Percentage (P)
Public toilets	1	0.28
Multi-family restrooms	73	20.33
Individual residence restrooms	266	74.09
>1 restroom home	19	5.29

Age group and religion

Religious affiliation varied among age groups, with Hinduism predominant across all brackets. In the 10-12 age group, there were 15 Hindus, 40 Christians, and 19 Muslims. For ages 13-15, Hindu participants comprised the majority (232), followed by Christians (20) and Muslims (17). Ages 16-18 saw 20 Hindus, four Christians, and four Muslims. A significant association between age group and religion was observed (χ² = 186.4, p < 0.001), with a strong correlation indicated by Cramer's V value of 0.572.

Age group and family type

Nuclear families were prevalent across all age groups, with the highest occurrence among ages 13-15. Joint families were less common but still notable in all age groups. The Chi-square test revealed a significant association between age group and family type (χ² = 24.3, p < 0.001), with Cramer's V value of 0.293 indicating a strong relationship.

Assessment of participants' knowledge improvement

Pre-intervention mean knowledge score was 5, rising significantly to 7.8 post-intervention, indicating substantial improvement. Chi-square tests demonstrated significant relationships between age group and religion, sources of MH information, mothers' educational status, and the availability of sanitization facilities (all p < 0.001). Cramer's V values ranged from 0.415 to 0.476, indicating moderate to strong correlations between age groups and these factors. These findings highlight the complex interplay between demographic factors and adolescent MH-related behaviors.

## Discussion

Understanding and managing menstrual hygiene is crucial for women's health, especially during adolescence, when menstruation begins. Our research aimed to analyze demographic characteristics, sources of knowledge, access to sanitation, and the effectiveness of interventions in improving menstrual health among adolescent females.

Most participants fell within the age range of 13 to 15, aligning with the global trend of menstruation onset during early adolescence. Additionally, Hindu affiliation predominated, reflecting local demographics. Cultural and religious beliefs significantly influenced menstrual health practices, highlighting the necessity of culturally sensitive interventions. Family composition, primarily nuclear, also impacted menstrual health routines, emphasizing the need for tailored interventions for diverse family structures [1-3].

Guidance from mothers emerged as a pivotal information source concerning menstrual hygiene. Peer-led programs and teachers played significant roles in combating misinformation and educating adolescents about menstrual health. The increasing influence of social media on knowledge acquisition underscores the importance of digital literacy to discern reliable information [4-6] (see Table 8 in Appendix).

Access to sanitation facilities is crucial for promoting menstrual well-being and human dignity. While private home bathrooms were common, challenges arose with multi-family restrooms, necessitating improvements in cleanliness and privacy. The insufficiency of public toilets highlights the importance of investing in equitable sanitation infrastructure [7].

Interventions effectively enhanced participants' understanding of menstrual hygiene, emphasizing the efficacy of inclusive and culturally sensitive approaches. Hands-on training and accessible resources addressed barriers to menstrual health management, bolstering individuals' confidence. Statistical analyses revealed significant correlations between demographic factors and menstrual health awareness, highlighting the importance of tailored interventions [8,9].

The study underscores the need for targeted interventions considering various demographic factors, including age, religion, family structure, and information preferences. By tailoring approaches to these aspects, we can empower adolescents to adopt healthy menstrual hygiene practices effectively. Understanding the unique traits and communication methods associated with different demographic groups is essential for designing impactful interventions that address the diverse needs of adolescent females [10].

Limitations

One limitation of our study is the reliance on self-reported data, which may be subject to recall bias. Additionally, the study focused exclusively on urban schools in Pallavaram taluk, limiting the generalizability of our findings to rural areas or other regions. Future research could explore these aspects to provide a more comprehensive understanding of menstrual hygiene practices among adolescent females.

## Conclusions

Our study sheds light on menstrual hygiene behaviors among adolescent school girls. To enhance credibility, we will standardize terminology and provide detailed methodological explanations. We acknowledge the need for a robust discussion on implications and limitations to guide future research and interventions. By addressing these aspects, we aim to contribute significantly to the advancement of menstrual health hygiene knowledge. Future directions include exploring rural settings and expanding interventions tailored to diverse demographic factors. Through evidence-based approaches, we strive to improve menstrual hygiene practices and overall well-being among adolescent girls.

## Appendices

**Table 8:** Assessment of knowledge, attitude, and behavior on menstrual hygiene among school-going adolescent girls. The data presented in the table is derived from References [4-8].

Socio-economic details	
Age (years)	
Religion	Hindu/Christian/Muslim/Others
Type of family	Joint family/nuclear family
Socioeconomic status	Upper class/upper middle/middle class/lower middle/lower class
Education qualification of subject	6/7/8/9/10/11/12th standard
Education qualification of mother	School/college/illiterate
Educational qualification of father	School/college/illiterate
Menstruation and menstrual hygiene knowledge among study participants	
1. What is menarche?	First periods/last periods/don’t know
2. Have you heard about menstruation (periods)?	Yes/no
3. From whom did you learn about menstruation?	Mother/sisters/friends/teachers
4. What is the normal age of attaining menstruation (periods)?	10-14 years/15-18 years
5. What is menstruation (periods)?	Regular physiological process/disease/sin/do not know
6. What is the cause of menstruation (periods)?	Hormones/diseases/do not know
7. Which do you think is the ideal absorbent during menstruation (periods)?	Sanitary napkins/new cloth/old cloth
8. What is the normal duration of menstruation (periods)?	3-7 days/10-15 days/15-20 days
9. Have you heard about menstrual hygiene?	Yes/No
10. Do poor menstrual hygiene practices lead to infections?	Yes/No
Menstruation and menstrual hygiene attitude among subjects	
1. Menstruation (periods) is annoying	Strongly agree/agree/neutral/disagree/strongly disagree
2. Menstruation (periods) is an external indication of women’s health	Strongly agree/agree/neutral/disagree/strongly disagree
3. Menstruation (periods) affects usual activities	Strongly agree/agree/neutral/disagree/strongly disagree
4. Menstruation (periods) is dirty	Strongly agree/agree/neutral/disagree/strongly disagree
5. During menstruation (periods) we should not touch holy books	Strongly agree/agree/neutral/disagree/strongly disagree
6. During menstruation (periods) we should not enter temples	Strongly agree/agree/neutral/disagree/strongly disagree
7. If we don’t take care of hygiene during menstruation (periods) it will cause disease	Strongly agree/agree/neutral/disagree/strongly disagree
8. Menstrual hygiene is very important	Strongly agree/agree/neutral/disagree/strongly disagree
9. Should take regular baths during menstruation (periods)	Strongly agree/agree/neutral/disagree/strongly disagree
10. Should change pads regularly during menstruation (periods)	Strongly agree/agree/neutral/disagree/strongly disagree
Menstruation and menstrual hygiene behavior among subjects	
1. What absorbent do you use during menstruation (periods)?	Sanitary pad/new cloth/old cloth
2. How frequently do you change pad/cloth per day?	Once in four hours/once in six hours/once in eight hours
3. Do you change pad or cloth in schools?	Yes/no
4. If you are using cloth or absorbent (re-usable), how do you dry it?	Outside room in sunlight/inside room with sunlight/without sunlight/not using reusable absorbent
5. How do you dispose your sanitary pads?	Buried/burned/dustbin/latrine/throw on road
6. When do you clean your genitalia?	Every time use toilet/during bathing/do not clean regularly
7. Material used for cleaning of external genitalia?	Water and antiseptic/soap and water/only water/not cleaning regularly
8. Have you missed your school due to menstruation?	Yes/no
9. Frequency of changing undergarments during menstruation?	Once a day/twice a day/alternate day
10. Before disposal do you wrap the solid absorbent?	Yes/no
